# Composite lipid-inflammatory indices and mortality risk in NHANES 2007–2016

**DOI:** 10.1097/MD.0000000000046630

**Published:** 2025-12-12

**Authors:** Xinyue Zhao, Nan Zhang, Tao Yan, Ziying Zhang, Lehui Li, Ning Cao, Jiwen Yang, Xingguang Zhang

**Affiliations:** a Department of Health Statistics, School of Public Health, Inner Mongolia Medical University, Hohhot, China; b Department of Environmental and Occupational Health, School of Public Health, Inner Mongolia Medical University, Hohhot, China; c Department of Pharmacology, College of Basic Medical Sciences, Inner Mongolia Medical University, Hohhot, China; d Department of Environmental Health, School of Public Health, Inner Mongolia Medical University, Hohhot, China; e Department of Epidemiology, School of Public Health, Inner Mongolia Medical University, Hohhot, China; f Neurosurgery Department, The Affiliated Hospital of Inner Mongolia Medical University, Hohhot, China.

**Keywords:** atherogenic index of plasma, cardiovascular disease, monocyte-to-high-density lipoprotein cholesterol ratio, neutrophil-to-high-density lipoprotein cholesterol ratio, systemic immune-inflammatory index

## Abstract

This study aims to investigate the associations of atherogenic index of plasma (AIP), systemic immune-inflammatory index (SII), monocyte-to-high-density lipoprotein cholesterol ratio (MHR), and neutrophil-to-high-density lipoprotein cholesterol ratio (NHR) with cardiovascular disease (CVD) mortality and all-cause mortality. Utilizing data from the National Health and Nutrition Examination Survey 2007–2016, Cox proportional hazards models were employed to evaluate the relationships between these biomarkers and mortality outcomes. Analysis of 11,188 participants based on Framingham Risk Scores revealed that higher AIP, SII, NHR, and MHR levels are significant risk factors for cardiovascular and all-cause mortality. These associations remained robust after adjusting for covariates including age, sex, race, smoking, and alcohol consumption. Similar trends were observed for all-cause mortality. AIP, SII, MHR, and NHR are associated with an increased risk of CVD and all-cause mortality, suggesting their potential utility as novel biomarkers for risk stratification in clinical practice.

## 1. Introduction

Cardiovascular diseases (CVDs) represent a global health crisis as the leading cause of mortality worldwide. Current WHO statistics indicate CVDs claim approximately 18 million lives annually, constituting 32% of total global deaths, with both incidence and mortality rates demonstrating a persistent upward trajectory. Notably, data from the Global Burden of Disease Study reveal a concerning rise in age-standardized CVD incidence rates in China, increasing from 646.2 to 652.2 per 100,000 person-years among individuals aged 1 to 79 years between 1990 and 2019.^[1]^

The development of effective prevention strategies hinges on early identification of high-risk populations and comprehensive understanding of risk factors.^[2]^ While dyslipidemia remains a well-established CVD risk factor, with Low-Density Lipoprotein Cholesterol (LDL-C) recognized as a primary driver of atherosclerosis pathogenesis, emerging evidence highlights the importance of novel composite biomarkers. The Atherogenic Index of Plasma (AIP), Neutrophil-to-HDL-C Ratio (NHR), Monocyte-to-HDL-C Ratio (MHR), and Systemic Immune-Inflammation Index (SII) have recently emerged as promising indicators that integrate both metabolic and inflammatory pathways.^[3–5]^ These biomarkers not only reflect lipid metabolism abnormalities but also capture systemic inflammatory states and immune activation, providing more comprehensive insights into CVD pathogenesis.

The critical role of inflammation in CVD progression is increasingly recognized. The SII, incorporating platelet, neutrophil and lymphocyte counts, offers a robust assessment of systemic inflammatory status.^[6–8]^ Clinical studies consistently demonstrate significant correlations between elevated SII levels and increased CVD risk.^[9,10]^ While HDL-C normally exhibits anti-inflammatory and antioxidant properties, inflammatory conditions may compromise its functionality. Elevated MHR and NHR values potentially reflect this HDL-C dysfunction, further exacerbating cardiovascular risk.^[11–13]^

This study utilizes data from the National Health and Nutrition Examination Survey (NHANES), which comprehensively evaluates the health and nutritional status of the non-institutionalized U.S. population. NHANES provides extensive demographic, clinical, and laboratory data, with detailed methodology documented in previous publications.^[14]^ While the individual associations of AIP, SII, NHR, and MHR with health outcomes have been explored separately, a critical gap exists in the literature regarding their combined evaluation. Our research directly addresses this gap by being the first to investigate all 4 indices simultaneously, thereby offering a novel and holistic view of their independent and comparative roles in risk prediction. Our analysis aims to elucidate whether AIP, SII, MHR, and NHR are independently associated with cardiovascular and all-cause mortality in a representative U.S. cohort. This study may provide new insights into the early prevention, diagnosis, and treatment of CVD-related mortality and all-cause mortality, as well as identify potential biomarkers.

## 2. Materials and methods

### 2.1. Study population

The data for this study were obtained from the publicly available NHANES 2007–2016 database, a nationally representative cross-sectional survey designed to assess the health and nutritional status of the U.S. population. All NHANES data are de-identified, publicly accessible, and approved by the National Center for Health Statistics (NCHS). Survival status was determined by linking participant records to the NCHS mortality database, establishing a longitudinal follow-up cohort. The NCHS Research Ethics Review Board approved the original survey protocol, and written informed consent was provided by all participants. Therefore, no additional ethical approval or participant consent was required for this analysis.

#### 2.1.1. *Inclusion criteria*

Aged ≥ 20 and < 80 years; complete Framingham risk score-related data, including age, sex, TC, high-density lipoprotein cholesterol (HDL-C), blood pressure, smoking status, and diabetes status; complete laboratory data for triglycerides (TG), TC, and complete blood count; availability of medical history data (hypertension, diabetes, coronary heart disease, heart failure, cancer) and follow-up records (survival status, follow-up duration).

#### 2.1.2. *Exclusion criteria*

Severe chronic diseases (e.g., malignancies, end-stage renal disease, advanced liver disease) that may affect lipid or inflammatory markers; individuals with missing data that substantially compromised analysis.

**Figure 1.**
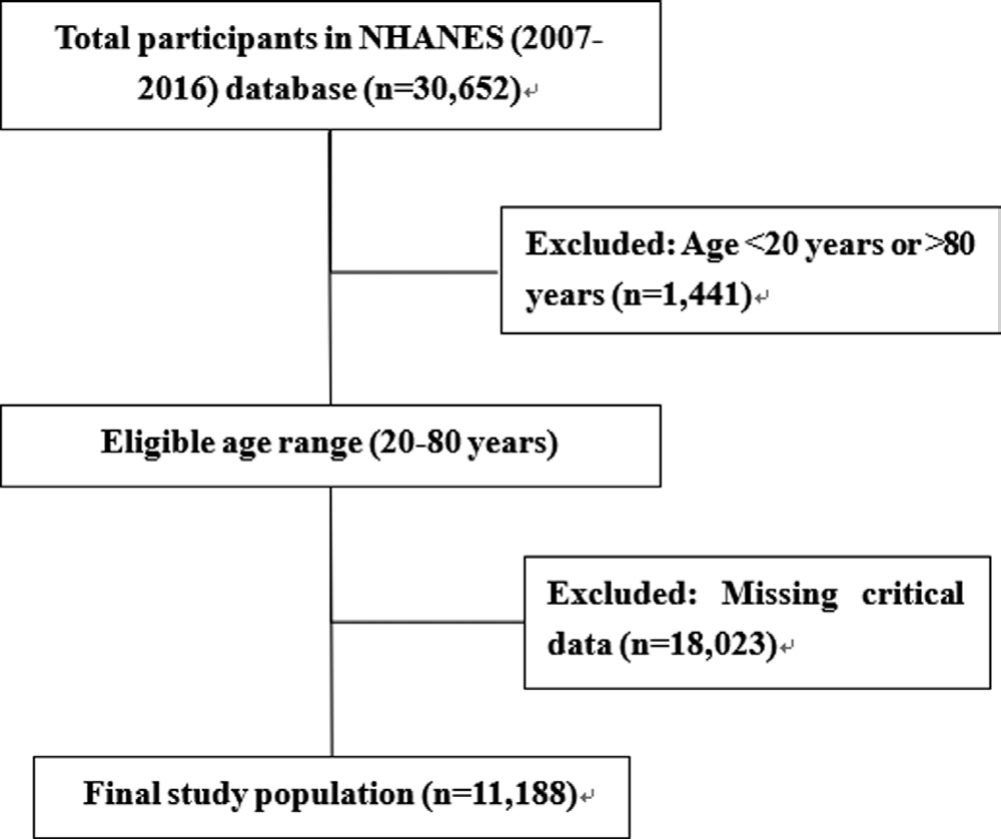
Flowchart of study population selection. After rigorous screening, a total of 11,188 eligible subjects were included in the final analysis. Regarding the handling of missing data, this study employed complete case analysis, where only participants with complete data for all analytical variables were retained for the final statistical analysis. The detailed screening process is illustrated in Figure 1.

After rigorous screening, a total of 11,188 eligible subjects were included in the final analysis. Regarding the handling of missing data, this study employed complete case analysis, where only participants with complete data for all analytical variables were retained for the final statistical analysis. The detailed screening process is illustrated in Figure 1.

### 2.2. Risk score calculation

Based on the database, risk scores were calculated by evaluating individual risk factors including age, sex, total cholesterol (TC), HDL-C, systolic blood pressure, and smoking status according to standardized risk assessment tables. The scores for each factor were summed to obtain a 10-year coronary heart disease risk score, which was categorized into 3 levels: low risk (<10%), intermediate risk (10%–20%), and high risk (>20%) to estimate an individual’s likelihood of developing cardiovascular disease.

### 2.3. Definitions

Diabetes was defined as prior diagnosis of diabetes; hypertension as prior diagnosis of hypertension; smoking history as having smoked at least 100 cigarettes in a lifetime; and alcohol consumption as intake of at least 12 alcoholic drinks of any type in the past year. Laboratory parameters obtained from NHANES included HDL-C, TG, and TC (mg/dL), as well as lymphocytes, neutrophils, monocytes, and platelets (measured in 1000 cells/μL). Derived indices were calculated as follows: AIP = log10(TG/HDL-C); Systemic Immune-Inflammation Index (SII) = (Neutrophils × Lymphocytes)/Platelets (with SII/100 used for analysis due to large numerical values); Neutrophil-to-HDL-C Ratio (NHR) = Neutrophils (1000 cells/μL)/HDL-C (mmol/L); and Monocyte-to-HDL-C Ratio (MHR) = Monocytes (1000 cells/μL)/HDL-C (mmol/L).

### 2.4. Statistical analysis

All analyses were performed using R software (version 4.3.3). Normally distributed continuous variables were expressed as mean ± standard deviation, while non-normally distributed variables were reported as median (interquartile range, IQR). Categorical variables were presented as frequency (percentage, %). Group comparisons were made using Student *t* test or Wilcoxon rank-sum test for continuous variables and Chi-square (*χ*^2^) test or Wilcoxon rank-sum test for categorical variables. Cox proportional hazards regression models were used to assess the associations of AIP, SII, MHR, and NHR (analyzed as both continuous and categorical variables) with cardiovascular mortality and all-cause mortality, adjusting for age (≤60 vs >60 years), sex, race/ethnicity, smoking status, alcohol consumption, hypertension status, and diabetes status. Results were expressed as hazard ratios (HR) with 95% confidence intervals, and a 2-sided *P*-value < 0.05 was considered statistically significant. Restricted cubic spline plots were generated using R packages including rms and ggplot.

## 3. Results

### 3.1. Baseline characteristics of the study population

The 11,188 enrolled participants were stratified by Framingham Risk Score into low-risk (n = 8391), intermediate-risk (n = 2354), and high-risk (n = 443) groups. The intermediate-risk group (mean age = 63) and high-risk group (mean age = 70) were significantly older than the low-risk group (mean age = 42). Both intermediate- and high-risk groups had higher proportions of male participants compared to the low-risk group. Significant differences (*P* < .001) were observed across all 3 groups for age, sex distribution, race/ethnicity, smoking status, alcohol consumption, and all investigated biomarkers (AIP, SII, MHR, NHR), as detailed in Table 1. Table S1, Supplemental Digital Content, https://links.lww.com/MD/Q932 includes additional details such as age group and race.

**Table 1:** Comparison of clinical characteristics among study participants stratified by cardiovascular risk groups

General information	Low-risk (n = 8391)	Intermediate-risk (n = 2354)	High-risk (n = 443)	*P*
Sex				
Male	3000 (36.75)	2027 (86.11)	376 (84.88)	<.001
Female	5391 (64.25)	327 (13.89)	67 (15.12)	
Age (year)	42 (30–54)	63 (55–70)	70 (59–76)	<.001
Smoking				
Yes	2941 (35.05)	1684 (71.54)	378 (85.33)	<.001
No	5450 (64.95)	670 (28.46)	65 (14.67)	
Drinking				
Yes	2200 (26.22)	1304 (55.40)	391 (88.26)	<.001
No	6191 (73.78)	1050 (44.60)	52 (11.74)	
Hypertension				
Yes	2200 (26.22)	1304 (55.40)	391 (88.26)	<.001
No	6191 (73.78)	1050 (44.60)	52 (11.74)	
Diabetes				
Yes	2200 (26.22)	1304 (55.40)	391 (88.26)	<.001
No	6191 (73.78)	1050 (44.60)	52 (11.74)	
HDL-C (mg/dl)	54 (44–65)	47 (40.5–56)	41 (36–47)	<.001
TG (mg/dl)	94 (66–138)	120 (85–171)	199 (167–236)	<.001
Lymphocytes (10³ cells/μL)	2 (1.6–2.4)	1.9 (1.5–2.3)	1.9 (1.5–2.4)	<.001
Neutrophils (10³ cells/μL)	3.6 (2.8–4.6)	3.9 (3–4.9)	4.3 (3.3–5.3)	<.001
Monocytes (10³ cells/μL)	0.5 (0.4–0.6)	0.5 (0.4–0.7)	0.6 (0.5–0.7)	<.001
Platelets (10³ cells/μL)	239 (205–283)	218 (185–260)	223 (185–267)	<.001
AIP	0.24 (0.04–0.46)	0.40 (0.21–0.60)	0.59 (0.38–0.75)	<.001
SII	434 (313–613)	451 (319–651)	489 (353–696)	<.001
NHR	2.61 (1.83–3.69)	3.11 (2.26–4.33)	4.03 (3.00–5.23)	<.001
MHR	0.35 (0.25–0.47)	0.44 (0.32–0.58)	0.54 (0.39–0.70)	<.001

Data in the table are expressed as number (%), mean ± standard deviation, or median (interquartile range).

AIP = atherogenic index of plasma, HDL-C = high-density lipoprotein cholesterol, MHR = monocyte-to-HDL-C ratio, NHR = neutrophil-to-HDL-C ratio, SII = systemic immune-inflammation index, TG = triglycerides.

### 3.2. Association of parameters with cardiovascular mortality

#### 3.2.1. Association of continuous variables with cardiovascular mortality

A multivariate Cox proportional hazards regression analysis was conducted in 236 cardiovascular disease patients, with mortality status (coded as: yes = 1, no = 0) as the dependent variable and AIP, SII/100, MHR, and NHR as independent variables. Three regression models were established: Model 1 (unadjusted); Model 2 (adjusted for age group [≤60 vs > 60 years], sex, and ethnicity); and Model 3 (further adjusted for smoking, alcohol consumption, hypertension, and diabetes). The results demonstrated that elevated levels of AIP, SII, MHR, and NHR were all significant risk factors for cardiovascular mortality (Table 2). Both AIP and MHR were statistically significant before adjustment, as well as after adjusting for age, sex, and race. However, while they remained risk factors after further adjustment for smoking, alcohol consumption, hypertension, and diabetes, they no longer showed statistical significance.

**Table 2:** Univariate and multivariate cox proportional hazards models of parameters with cardiovascular disease mortality.

Variable	Model 1		Model 2		Model 3	
	HR (95% CL)	*P*	HR (95% CL)	*P*	HR (95% CL)	*P*
AIP	1.97 (1.30–2.99)	.001	1.93 (1.24–3.01)	.004	1.23 (0.78–1.96)	.371
SII/100	1.10 (1.08–1.12)	<.001	1.10 (1.08–1.120)	<.001	1.09 (1.07–1.11)	<.001
NHR	1.19 (1.13–1.26)	<.001	1.19 (1.14–1.25)	<.001	1.15 (1.08–1.21)	<.001
MHR	2.72 (1.69–4.37)	<.001	2.24 (1.31–3.81)	<.003	1.49 (0.83–2.67)	.186

AIP = atherogenic index of plasma, MHR = monocyte-to-HDL-C ratio, NHR = neutrophil-to-HDL-C ratio, SII = systemic immune-inflammation index.

#### 3.2.2. Association of tertile groups of parameters with cardiovascular disease mortality

A multivariate Cox proportional hazards regression analysis was conducted in 236 cardiovascular disease patients, with mortality status (yes = 1, no = 0) as the dependent variable and tertile groups of AIP, SII/100, MHR, and NHR as independent variables. Three regression models were constructed: Model 1 (unadjusted); Model 2 (adjusted for age group [≤60 vs > 60 years], sex, and ethnicity); and Model 3 (further adjusted for smoking, alcohol consumption, hypertension, and diabetes). The results demonstrated that elevated tertiles of AIP, SII, MHR, and NHR were all associated with increased cardiovascular mortality risk, showing a significant dose-response relationship where higher parameter levels corresponded to progressively greater mortality risk (Table 3). When categorized by tertiles, both AIP and MHR were statistically significant before adjustment and after adjusting for age, sex, and race. However, after further adjustment for smoking, alcohol consumption, hypertension, and diabetes, they still showed an increasing trend but no longer exhibited statistical significance.

**Table 3:** Univariate and multivariate cox proportional hazards models of parameter tertiles with cardiovascular disease mortality.

Variable	group	Model 1		Model 2		Model 3	
		HR (95% CL)	*P*	HR (95% CL)	*P*	HR (95% CL)	*P*
AIP	Q1	1	.001	1	.003	1	.296
	Q2	1.39 (0.98–1.98)	.065	1.31 (0.92–1.87)	.137	1.16 (0.81–1.66)	.421
	Q3	1.90 (1.36–2.64)	<.001	1.78 (1.27–2.51)	.001	1.32 (0.93–1.87)	.123
SII/100	Q1	1	<.001	1	<.001	1	<.001
	Q2	0.91 (0.62–1.32)	.604	0.95 (0.65–1.38)	.791	0.87 (0.60–1.27)	.478
	Q3	2.07 (1.51–2.83)	<.001	2.19 (1.59–3.02)	<.001	1.95 (1.41–2.68)	<.001
NHR	Q1	1	<.001	1	<.001	1	<.001
	Q2	1.49 (1.04–2.14)	.032	1.63 (1.13–2.35)	.01	1.41 (0.97–2.04)	.070
	Q3	2.35 (1.68–3.28)	<.001	2.73 (1.93–3.87)	<.001	2.03 (1.42–2.91)	<.001
MHR	Q1	1	<.001	1	.007	1	.250
	Q2	1.51 (1.07–2.14)	.019	1.46 (1.03–2.08)	.034	1.27 (0.89–1.82)	.181
	Q3	1.96 (1.40–2.73)	<.001	1.76 (1.24–2.50)	.002	1.35 (0.94–1.93)	.104

AIP = atherogenic index of plasma, MHR = monocyte-to-HDL-C ratio, NHR = neutrophil-to-HDL-C ratio, SII = systemic immune-inflammation index.

#### 3.2.3. Association of parameters with cardiovascular disease across different risk strata

A stratified analysis was conducted in 236 cardiovascular disease patients categorized by baseline risk (≤10% vs > 10%). Using mortality status (yes = 1, no = 0) as the dependent variable and tertiles of AIP, SII, MHR, and NHR as independent variables, multivariate Cox proportional hazards regression analyses were performed. Results presented in Table S2, Supplemental Digital Content, https://links.lww.com/MD/Q932 show the associations after adjustment for sex, age group (≤60 vs > 60 years), and ethnicity. Notably, even in the lower-risk stratum (≤10%), AIP, SII, MHR, and NHR maintained significant predictive value for mortality risk. However, in the group with tertiles of AIP and MHR below 10%, both indices were risk factors but did not exhibit statistical significance (Table S2, Supplemental Digital Content).

#### 3.2.4. Restricted cubic spline analysis

**Figure 2.**
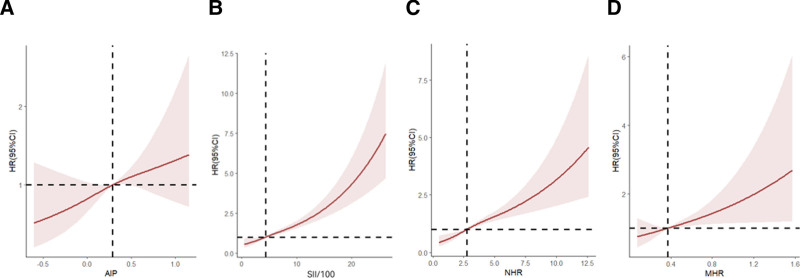
Restricted cubic spline curves of parameters with cardiovascular mortality risk. After adjusting for sex, age subgroups (≤60 yr, >60 yr), and race, the risk of death due to cardiovascular disease showed an increasing trend with increasing AIP, SII, MHR, and NHR. See figure 2. Note: *Restricted cubic spline curves for AIP, SII/100, NHR, MHR, adjusted for sex, age and race. Wald tests confirmed significant nonlinear associations for AIP (χ²=5.39, *P* = .067), SII/100 (χ²=81.98, *P* < .0001), NHR (χ²=42.55, *P* < .0001), MHR (χ²=9.09, *P* = .01). Shaded regions represent 95% confidence intervals. The x-axis (horizontal axis) represents the expression levels of each composite index, and the y-axis (vertical axis) represents the HR values. AIP = atherogenic index of plasma, HR = hazard ratio, MHR = monocyte-to-HDL-C ratio, NHR = neutrophil-to-HDL-C ratio, SII = systemic immune-inflammation index.

After adjusting for sex, age subgroups (≤60 years, >60 years), and race, the risk of death due to cardiovascular disease showed an increasing trend with increasing AIP, SII, MHR, and NHR. See Figure 2.

### 3.3. Association of parameters with all-cause mortality

#### 3.3.1. Association of continuous variables with all-cause mortality

The multifactorial Cox proportional risk regression model was analyzed with whether to die (assigned value: yes = 1, no = 0) as the dependent variable, and AIP, SII/100, MHR, and NHR as the independent variables, respectively, and the regression models were constructed: model 1: no other covariates were included; model 2: age grouping (≤60 years old, >60 years old), gender, and ethnicity were included as covariates on the basis of model 1; and model 3: Add smoking, alcohol consumption, hypertension, diabetes as covariates to model 2. The results found that high levels of AIP, SII, MHR, and NHR were risk factors, as shown in Table 4.

**Table 4:** Univariate and multivariate cox proportional hazards models of parameters with all-cause mortality.

Variable	Model 1		Model 2		Model 3	
	HR (95% CL)	*P*	HR (95% CL)	*P*	HR (95% CL)	*P*
AIP	1.69 (1.36–2.10)	<.001	1.53 (1.21–1.93)	<.001	1.05 (0.83–1.34)	.674
SII/100	1.02 (1.02–1.03)	<.001	1.03 (1.02–1.03)	<.001	1.02 (1.02–1.03)	<.001
NHR	1.04 (1.03–1.05)	<.001	1.05 (1.04–1.06)	<.001	1.04 (1.03–1.05)	<.001
MHR	1.33 (1.23–1.43)	<.001	1.18 (1.09–1.28)	<.001	1.18 (1.06–1.30)	.002

AIP = atherogenic index of plasma, MHR = monocyte-to-HDL-C ratio, NHR = neutrophil-to-HDL-C ratio, SII = systemic immune-inflammation index.

#### 3.3.2. Association of parameter tertiles with all-cause mortality

Multifactorial Cox proportional risk regression models were analyzed using whether to die (assignment: yes = 1, no = 0) as the dependent variable, and tertile groupings of AIP, SII/100, MHR, and NHR as the independent variables, and regression models were constructed as follows: Model 1: no other covariates were included; Model 2: age groupings (≤60 years, >60 years), gender, and race were added as covariates to Model 1; Model 3: smoking, alcohol consumption, hypertension, and diabetes were added as covariates to Model 2; and Model 4: smoking, alcohol consumption, hypertension, and diabetes were added to Model 2 as covariates. variables; Model 3: add smoking, alcohol consumption, hypertension, and diabetes as covariates based on Model 2. It was found that higher AIP, SII, MHR, and NHR levels were associated with increased mortality risk, as shown in Table 5.

**Table 5:** Univariate and multivariate cox proportional hazards models of parameter tertiles with all-cause mortality.

Variable	group	Model 1		Model 2		Model 3	
		HR (95% CL)	*P*	HR (95% CL)	*P*	HR (95% CL)	*P*
AIP	Q1	1	<.001	1	<.001	1	.544
	Q2	1.21 (1.01–1.45)	.035	1.11 (0.93–1.34)	.245	0.99 (0.83–1.19)	.938
	Q3	1.57 (1.32–1.85)	<.001	1.39 (1.17–1.66)	<.001	1.08 (0.90–1.29)	.411
SII/100	Q1	1	<.001	1	<.001	1	<.001
	Q2	0.99 (0.82–1.19)	.898	0.99 (0.83–1.20)	.945	0.93 (0.77–1.12)	.460
	Q3	1.67 (1.42–1.97)	<.001	1.64 (1.39–1.94)	<.001	1.46 (1.23–1.73)	<.001
NHR	Q1	1	<.001	1	<.001	1	<.001
	Q2	1.04 (0.87–1.25)	.67	1.10 (0.91–1.32)	.334	0.96 (0.80–1.16)	.699
	Q3	1.63 (1.39–1.92)	<.001	1.76 (1.49–2.09)	<.001	1.34 (1.12–1.60)	.001
MHR	Q1	1	<.001	1	<.001	1	.167
	Q2	1.14 (0.95–1.35)	.162	1.08 (0.9–1.29)	.416	0.97 (0.81–1.16)	.716
	Q3	1.60 (1.36–1.89)	<.001	1.42 (1.19–1.70)	<.001	1.12 (0.94–1.35)	.205

AIP = atherogenic index of plasma, MHR = monocyte-to-HDL-C ratio, NHR = neutrophil-to-HDL-C ratio, SII = systemic immune-inflammation index.

#### 3.3.3. Association of parameters with all-cause mortality across different risk strata

In the analysis of patients with all-cause mortality (11,188 cases), stratified by risk level (≤10% vs >10%), with mortality (assigned as: yes = 1, no = 0) as the dependent variable, and AIP, SII/100, MHR, and NHR divided into tertiles as independent variables, multivariate Cox proportional hazards regression models were performed. The results, adjusted for sex, age group (≤60 years, >60 years), and race, showed that even in the lower-risk stratum (≤10%), AIP, SII, MHR, and NHR remained significant associated with of mortality. See Table S3, Supplemental Digital Content, https://links.lww.com/MD/Q932 for details.

#### 3.3.4. Restricted cubic spline analysis

**Figure 3.**
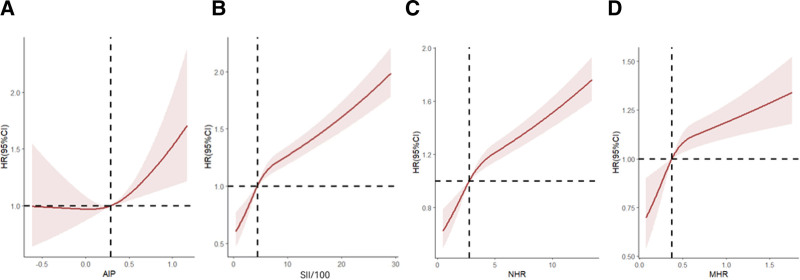
Restricted cubic spline curves showing the association between each parameter and mortality risk. After adjusting for sex, age group (≤60 yr, >60 yr), and race, the risk of mortality showed an increasing trend with rising levels of AIP, SII, MHR, and NHR. See figure 3. Note: *Restricted cubic spline curves for AIP, SII/100, NHR, MHR, adjusted for sex, age and race. Wald tests confirmed significant nonlinear associations for AIP (χ²=12.21, *P* < .01), SII/100 (χ²=156.65, *P* < .0001), NHR (χ²=150.74, *P* < .0001), MHR (χ²=21.86, *P* < .0001). Shaded regions represent 95% confidence intervals. The x-axis (horizontal axis) represents the expression levels of each composite index, and the y-axis (vertical axis) represents the HR values. AIP = atherogenic index of plasma, HR = hazard ratio, SII = systemic immune-inflammation index, NHR = neutrophil-to-HDL-C ratio, MHR = monocyte-to-HDL-C ratio.

After adjusting for sex, age group (≤60 years, >60 years), and race, the risk of mortality showed an increasing trend with rising levels of AIP, SII, MHR, and NHR. See Figure 3.

## 4. Discussion

This study analyzed the NHANES database (2007–2016) to explore the associations of AIP, SII, MHR, and NHR with cardiovascular mortality and all-cause mortality. The results show that AIP, SII, MHR, and NHR are significant risk factors for both cardiovascular and all-cause mortality, providing new scientific evidence for cardiovascular risk assessment and stratification. These findings are crucial for the early identification of high-risk populations and have important public health and clinical implications.

AIP has been identified as a risk factor for cardiovascular disease. Elevated AIP levels indicate a more atherogenic lipid profile, reflecting an imbalance between pro-atherogenic and anti-atherogenic lipoproteins, which may promote the formation of atherosclerotic plaques. Our study confirms that AIP is associated with an increased risk of mortality, which is consistent with previous reports,^[14–16]^ highlighting its potential role in the pathogenesis of cardiovascular diseases. Similarly, MHR, NHR, and SII were significantly associated with a higher mortality risk in cardiovascular patients, which is in line with previous literature.^[17–19]^ The loss of statistical significance for AIP in Model 3 of our results may be attributed to the fact that smoking, alcohol consumption, hypertension, and diabetes are well-established confounding factors that share common pathological pathways (such as chronic inflammation, endothelial dysfunction, and lipid metabolism disorders) with AIP. Including these variables in the model may have accounted for the overlapping variance between AIP and mortality risk, thereby attenuating the independent associative strength of AIP.

Monocytes and neutrophils play a key role in inflammatory responses.^[20]^ Elevated MHR and NHR may indicate enhanced systemic inflammation. Monocytes can differentiate into macrophages in the arterial intima, engulf lipids to form foam cells, and accelerate the progression of atherosclerotic plaques. Neutrophils release various inflammatory mediators, further contributing to the inflammatory cascade in atherosclerosis.^[21–23]^ In this study, the fully adjusted model’s additional variables (smoking, alcohol, hypertension, diabetes) are well-validated confounders sharing pathophysiological links with MHR – smoking raises MHR via inflammation/HDL-C reduction, while hypertension/diabetes disrupt monocyte/HDL-C function, overlapping with MHR’s “inflammatory-lipid imbalance.” Including them isolates shared mortality risk variance, weakening MHR’s independent association below statistical significance. SII reflects the systemic immune-inflammatory status, but its relationship with cardiovascular disease may be more complex and influenced by other intrinsic factors.^[24]^ Our study’s findings differ from Drwila et al: AIP was not an MACE predictor in their young-old subgroup, and while it was significant in the old-old subgroup, it showed a negative predictive effect. This discrepancy may stem from their specific population (only elderly non-ST-segment elevation myocardial infarction patients) and short 1-year follow-up – unlike our broader cohort and longer follow-up.^[25]^

This cohort study has strong external validity, which enhances the generalizability of its conclusions. However, as an observational study, potential confounding biases due to population imbalances cannot be excluded. To mitigate this, covariates such as age, sex, race, smoking, and alcohol consumption were adjusted using multivariate Cox regression analysis, improving the reliability of the results. This study has several limitations inherent in its retrospective design: although the NHANES database provides nationally representative data in the United States, its generalizability to other ethnicities and regions needs further validation. In addition, the observational nature of the analysis precludes causal inferences, and future mechanistic studies through in vitro and animal experiments are necessary. Potential residual confounding may also exist due to unmeasured variables such as diet, physical activity, and stress levels, which should be addressed in subsequent research.

Notably, this study has important implications for the prevention of cardiovascular disease (CVD) in China, where the increasing morbidity and mortality of CVD pose a critical public health challenge.^[1]^ AIP, SII, MHR, and NHR, as potential risk markers, could provide new approaches for risk stratification in the Chinese population. Given the differences in diet, lifestyle, and genetic background between Chinese and U.S. cohorts, prospective studies in China are needed to validate these biomarkers and develop population-specific risk models. Clinically, these indices may help in the early identification of high-risk individuals, enabling personalized prevention strategies to reduce the burden of CVD.

In conclusion, this study demonstrates that AIP, SII, MHR, and NHR are all associated with CVD mortality and all-cause mortality. These indices may be useful markers for risk stratification of CVD mortality and all-cause mortality, even in populations with low traditional risk profiles. These findings provide valuable references for developing preventive strategies for CVD, prevention and control protocols for mortality risk, and targeted therapeutic interventions.

## Author contributions

**Data curation:** Xinyue Zhao.

**Formal analysis:** Xinyue Zhao, Lehui Li.

**Methodology:** Xinyue Zhao, Tao Yan, Ziying Zhang, Lehui Li, Ning Cao, Jiwen Yang, Xingguang Zhang.

**Resources:** Nan Zhang, Tao Yan, Jiwen Yang, Xingguang Zhang.

**Writing – original draft:** Xinyue Zhao.

**Writing – review & editing:** Xinyue Zhao, Nan Zhang, Xingguang Zhang.

## Supplementary Material
