# Supplementary material for: Composite lipid-inflammatory indices and mortality risk in NHANES 2007–2016
Source: Medicine (Baltimore). 2025 Dec 12;104(50):e46630. doi: 10.1097/MD.0000000000046630 (PMC12708090; doi:10.1097/MD.0000000000046630)
Supplement: Supplementary file 1 [file medi-104-e46630-s001.docx]

Table S1 Comparison of Clinical Characteristics Among Study Participants Stratified by Cardiovascular Risk Groups

| General Information | Low-Risk (n=8,391) | Intermediate-Risk (n=2,354) | High-Risk (n=443) | *P* |
| --- | --- | --- | --- | --- |
| Sex |  |  |  |  |
| male | 3000（36.75） | 2027（86.11） | 376（84.88） | <0.001 |
| female | 5391（64.25） | 327（13.89） | 67（15.12） |  |
| Age (years) | 42（30~54） | 63（55~70） | 70（59~76） | <0.001 |
| Age group |  |  |  |  |
| 20-39 years | 3775(44.99） | 87（3.70） | 5（1.13） | <0.001 |
| 40-49 years | 1767（21.06） | 254（10.79） | 25（5.64） |  |
| 50-59 years | 1327（15.81） | 548（23.28） | 89（20.09） |  |
| 60-69 years | 1096（13.06） | 809（34.37） | 97（21.90） |  |
| 70-79 years | 426（5.08） | 656（27.87） | 227（51.24） |  |
| Race |  |  |  |  |
| Mexican American | 1407（16.77） | 339（14.40） | 50（11.29） | <0.001 |
| Hispanic | 968（11.54） | 291（12.36） | 46（10.38） |  |
| Non-Hispanic White | 3295（39.27） | 1050（44.60） | 226（51.02） |  |
| Non-Hispanic Black | 1706（20.33） | 476（20.22） | 88(19.86) |  |
| other | 1015（12.10） | 198（8.41） | 33（7.45） |  |
| Smoking |  |  |  |  |
| Yes | 2941（35.05） | 1684（71.54） | 378（85.33） | <0.001 |
| No | 5450（64.95） | 670（28.46） | 65（14.67） |  |
| Drinking |  |  |  |  |
| Yes | 2200（26.22） | 1304（55.40） | 391（88.26） | <0.001 |
| No | 6191（73.78） | 1050（44.60） | 52（11.74） |  |
| Hypertension |  |  |  |  |
| Yes | 2200（26.22） | 1304（55.40） | 391（88.26） | <0.001 |
| No | 6191（73.78） | 1050（44.60） | 52（11.74） |  |
| Diabetes |  |  |  |  |
| Yes | 2200（26.22） | 1304（55.40） | 391（88.26） | <0.001 |
| No | 6191（73.78） | 1050（44.60） | 52（11.74） |  |
| HDL-C（mg/dl） | 54（44~65） | 47（40.5~56） | 41（36~47） | <0.001 |
| TG（mg/dl） | 94（66~138） | 120（85~171） | 199（167~236） | <0.001 |
| **Lymphocytes (10³ cells/μL)** | 2（1.6~2.4） | 1.9（1.5~2.3） | 1.9（1.5~2.4） | <0.001 |
| **Neutrophils (10³ cells/μL)** | 3.6（2.8~4.6） | 3.9（3~4.9) | 4.3（3.3~5.3） | <0.001 |
| **Monocytes (10³ cells/μL)** | 0.5（0.4~0.6） | 0.5（0.4~0.7） | 0.6（0.5~0.7） | <0.001 |
| **Platelets (10³ cells/μL)** | 239（205~283） | 218（185~260） | 223（185~267） | <0.001 |
| AIP | 0.24（0.04~0.46） | 0.40（0.21~0.60） | 0.59（0.38~0.75） | <0.001 |
| SII | 434（313~613） | 451（319~651） | 489（353~696） | <0.001 |
| NHR | 2.61（1.83~3.69） | 3.11（2.26~4.33） | 4.03（3.00~5.23） | <0.001 |
| MHR | 0.35（0.25~0.47） | 0.44（0.32~0.58） | 0.54（0.39~0.70） | <0.001 |

Note: Data in the table are expressed as number (%), mean±standard deviation, or median (interquartile range).

HDL-C, high-density lipoprotein cholesterol; TG, triglycerides; AIP, Atherogenic Index of Plasma; SII, Systemic Immune-Inflammation Index; NHR, Neutrophil-to-HDL-C Ratio; MHR, Monocyte-to-HDL-C Ratio.

| Table S2 Univariate and Multivariate Cox Proportional Hazards Models of Parameter Tertiles with Cardiovascular Disease Mortality Stratified by Baseline Risk | | | | | |
| --- | --- | --- | --- | --- | --- |
| Variable | group | ≤10% | | >10% | |
|  |  | *HR*（95%CL） | *P* | *HR*（95%CL） | *P* |
| AIP | Q1 | 1 | 0.104 | 1 | 0.456 |
|  | Q2 | 1.11(0.64~1.943) | 0.706 | 1.22(0.75~1.97) | 0.428 |
|  | Q3 | 1.76(1.01~3.08) | 0.045 | 1.34(0.84~2.13) | 0.213 |
| SII/100 | Q1 | 1 | 0.004 | 1 | <0.001 |
|  | Q2 | 1.53(0.82~2.84) | 0.183 | 0.71(0.44~1.15) | 0.166 |
|  | Q3 | 2.61(1.46~4.66) | 0.001 | 1.86(1.27~2.74) | 0.002 |
| NHR | Q1 | 1 | <0.001 | 1 | 0.003 |
|  | Q2 | 1.53(0.86~2.72) | 0.146 | 1.49(0.91~2.45) | 0.112 |
|  | Q3 | 3.15(1.80~5.50) | <0.001 | 2.14(1.34~3.42) | 0.001 |
| MHR | Q1 | 1 | 0.130 | 1 | 0.315 |
|  | Q2 | 1.52(0.90~2.58) | 0.118 | 1.30(0.81~2.11) | 0.278 |
|  | Q3 | 1.76(0.98~3.17) | 0.060 | 1.43(0.90~2.27) | 0.129 |

Note: AIP, Atherogenic Index of Plasma; SII, Systemic Immune-Inflammation Index; NHR, Neutrophil-to-HDL-C Ratio; MHR, Monocyte-to-HDL-C Ratio.

| Table S3 Univariate and Multivariate Cox Proportional Hazards Models of Parameters and All-Cause Mortality Across Different Risk Strata | | | | | |
| --- | --- | --- | --- | --- | --- |
| Variable | group | ≤10% | | >10% | |
|  |  | *HR*（95%CL） | *P* | *HR*（95%CL） | *P* |
| AIP | Q1 | 1 | 0.013 | 1 | 0.560 |
|  | Q2 | 1.18(0.91~1.54) | 0.205 | 0.88(0.68~1.14) | 0.331 |
|  | Q3 | 1.49(1.14~1.95) | 0.003 | 0.96(0.76~1.23) | 0.756 |
| SII/100 | Q1 | 1 | 0.004 | 1 | <0.001 |
|  | Q2 | 1.10(0.83~1.46) | 0.496 | 0.89(0.69~1.14) | 0.356 |
|  | Q3 | 1.52(1.17~1.98) | 0.002 | 1.59(1.28~1.98) | <0.001 |
| NHR | Q1 | 1 | <0.001 | 1 | <0.001 |
|  | Q2 | 1.19(0.92~1.55) | 0.189 | 0.91(0.70~1.17) | 0.456 |
|  | Q3 | 1.77(1.36~2.31) | <0.001 | 1.42(1.13~1.80) | 0.003 |
| MHR | Q1 | 1 | 0.011 | 1 | 0.196 |
|  | Q2 | 1.09(0.84~1.41) | 0.509 | 0.95(0.73~1.22) | 0.674 |
|  | Q3 | 1.50(1.14~1.96) | 0.004 | 1.13(0.89~1.44) | 0.314 |

Note: AIP, Atherogenic Index of Plasma; SII, Systemic Immune-Inflammation Index; NHR, Neutrophil-to-HDL-C Ratio; MHR, Monocyte-to-HDL-C Ratio.
